# Supplementary material for: Automated device for continuous stirring while sampling in liquid chromatography systems
Source: Commun Chem. 2020 Dec 3;3:180. doi: 10.1038/s42004-020-00427-5 (PMC9814086; doi:10.1038/s42004-020-00427-5)
Supplement: Supplementary file 1 — Supplementary Information [file 42004_2020_427_MOESM1_ESM.pdf]

Supplementary information: Automated device for continuous stirring while sampling in liquid chromatography systems

Omer Markovitch[1,2,*], Jim Ottelé[2], Obe Veldman[3] and Sijbren Otto[2]
1 Origins Center, Groningen, The Netherlands
2 Center for Systems Chemistry, Stratingh Institute, University of Groningen, Groningen, The Netherlands
3 Veldman Technische Ontwikkeling en Advisering, Groningen, The Netherlands
* omermar@gmail.com

Supplementary Note 1. UPLC eluents and gradient

Both H_2O and acetonitrile (CH_3CN) used were UPLC grade and contain 0.1 v% trifluoroacetic acid (CF_3CO_2H). Gradient is given in Table S1.

Table S1: UPLC gradient.

Time [min.]	% H_2O	% Acetonitrile
0.0	90	10
1.0	90	10
1.3	75	25
3.0	72	28
11.0	60	40
11.5	5	95
12.0	5	95
12.5	90	10
17.0	90	10

Supplementary Note 2. Peak integration algorithm

Waters Empower software was used for the integration of peaks. The ApexTrack algorithm was used with the following parameters: Start=5.9 (min.), End=12.5 (min.), Peak Width=4.65 (sec.), Detection Threshold=24, Liftoff=0.0%, Touchdown=0.5%, Minimum Area=9000 and Minimum Height=8000.

Supplementary Note 3. Chromatograms

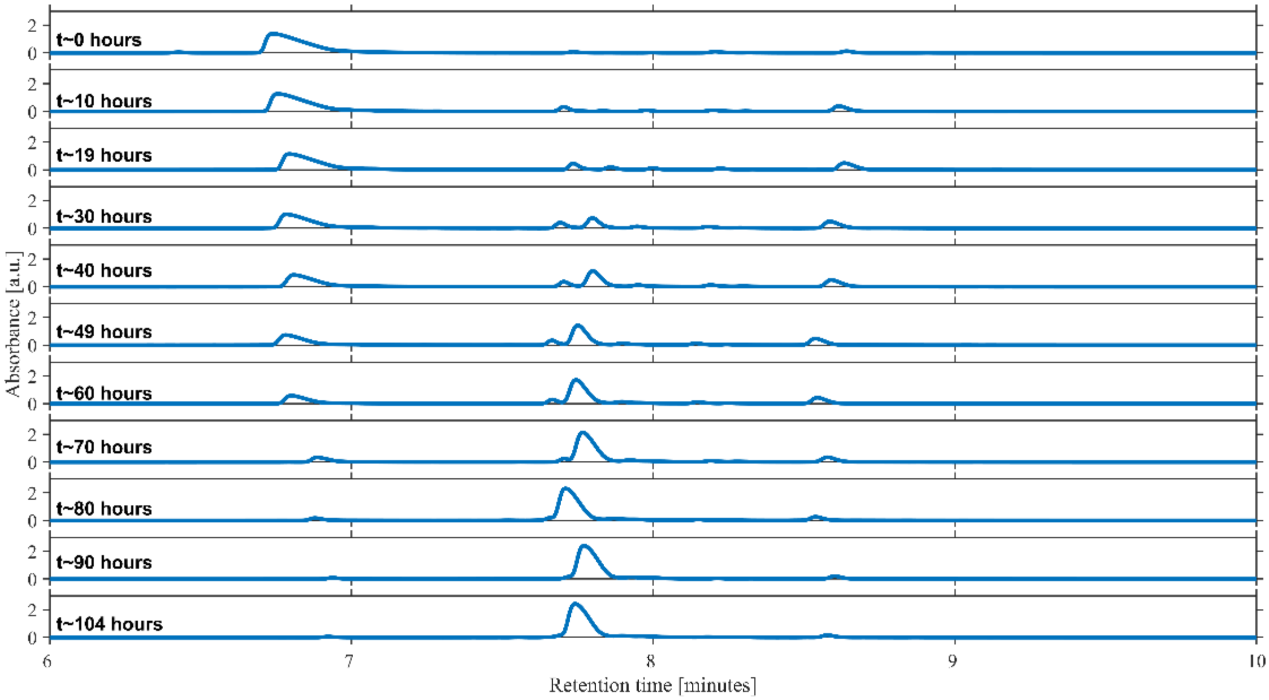

Figure S1: UPLC chromatograms of a sample measured under continuous stirring at 1000 rpm ("1000 rpm, 1" in Fig. 3a in the main text).

Supplementary Note 4. Quantitative validation using video analysis

In order to further validate the stirring device and provide a quantitative assessment of its stirring, a video analysis has been performed on the individual positions. In this analysis, a stirred sample is recorded using a high speed camera for a short duration and then the video is analysed by counting the number of revolutions the Teflon-coated magnet has undergone when the device is set at 200 or 1000 rpm. Relative to the user set speed, the average speed of rotation is 0.99 and 0.98 respectively for 200 and 1000 rpm (Table S2), thus confirming the device's functionality. Because of symmetry the analysis focused on the positions in one quadrant surrounding the central motor and in addition all the positions used in the present study. Results are summarized in Table S2. Videos are available as an attachment (note: attached videos are slowed down ×4).

Table S2: Measured rpm of positions (see Fig. 1 in the main text) analysed by video. Analysis counted 30-40 revolutions per position, and data is given in revolutions per minute (rpm). Videos were taken using a Sony actioncam AS20 camera at 120 frames per second (fps), and manually analysed by visual inspection using Kinovea software.

Position	Device 200 rpm	Device 1000 rpm
A4	201	986
A5	198	962
C2	196	1011
D2	197	986
E2	200	974
F2	197	986
F4	202	968
F5	197	968
C7	198	986
D7	195	968

Supplementary Note 5. Reproducing the stirring device

The components are (in brackets is the component 3D model file name):

a) Disc onto which magnets will be attached to create the magnetic field (disc.stl; https://www.supermagnete.nl/eng/block-magnets-neodymium/block-magnet-25mm-8mm-1mm_Q-25-08-01-N). This disc may be 3D printed with ABS material (235 °C printing temperature and 90 °C print bed temperature).
b) A CD/DVD Motor (MABUCHI motor RF300FA).
c) Modified sample holder (holder.stl). This design allows for improved visual inspection of some of the sample position. The device's profile is approximately 4 mm higher than the standard default sample holder, and consequently the UPLC needle height should be adjusted.
d) Cover for disc (coverdisc.stl).
e) Sensor to detect the disc speed and sensor housing (infineon TLE4905L; sensor.stl).
f) Arduino firmware program – attached.
g) Arduino microcontroller (Arduino Pro Mini 3.3V 8MHz).
h) Printed circuit board – see below.

i) Display (SSD1306 OLED display 1 inch I2C) and part for mounting the display onto the control device (display.stl).
j) Power switch.
k) Knob to adjust stirring speed (knob.stl).
l) Housing for AA batteries.
m) Control device in the shape of a standard sample holder (control.stl).
n) Cover for control device (covercontrol.stl).
o) Cover for motor (covermotor.stl).

Figure S2 provides snapshots of the 3D components. All of the 3D design files of the components are also available from the corresponding author and at: https://zenodo.org/record/4118046.

Device's assembly steps:

1. Make holes for magnets in disc (component a), and attach magnets. It is possible to add more magnets (for example, 2 in each side) for a stronger magnetic field. Make sure the disc is balanced.
2. Connect disc to motor (component b).
3. Place disc-motor within the modified sample holder (component c). Make sure motor is firmly attached to the modified sample holder. Rubber band may be needed.
4. Attach disc cover to the bottom of the modified sample holder (component d). Screws are needed.
5. Install speed sensor (component e) near the motor.
6. Install program onto Arduino controller (components f & g).
7. Connect circuit board (component h) to display, power switch, speed knob, speed sensor, battery housing and Arduino (components i, j, k, l & h). Cables and soldering are needed.
   7.1. Place the connected circuit board inside the control device (component m).
   7.2. Attach cover for control device (component n).
8. Attach cover for motor (component o).

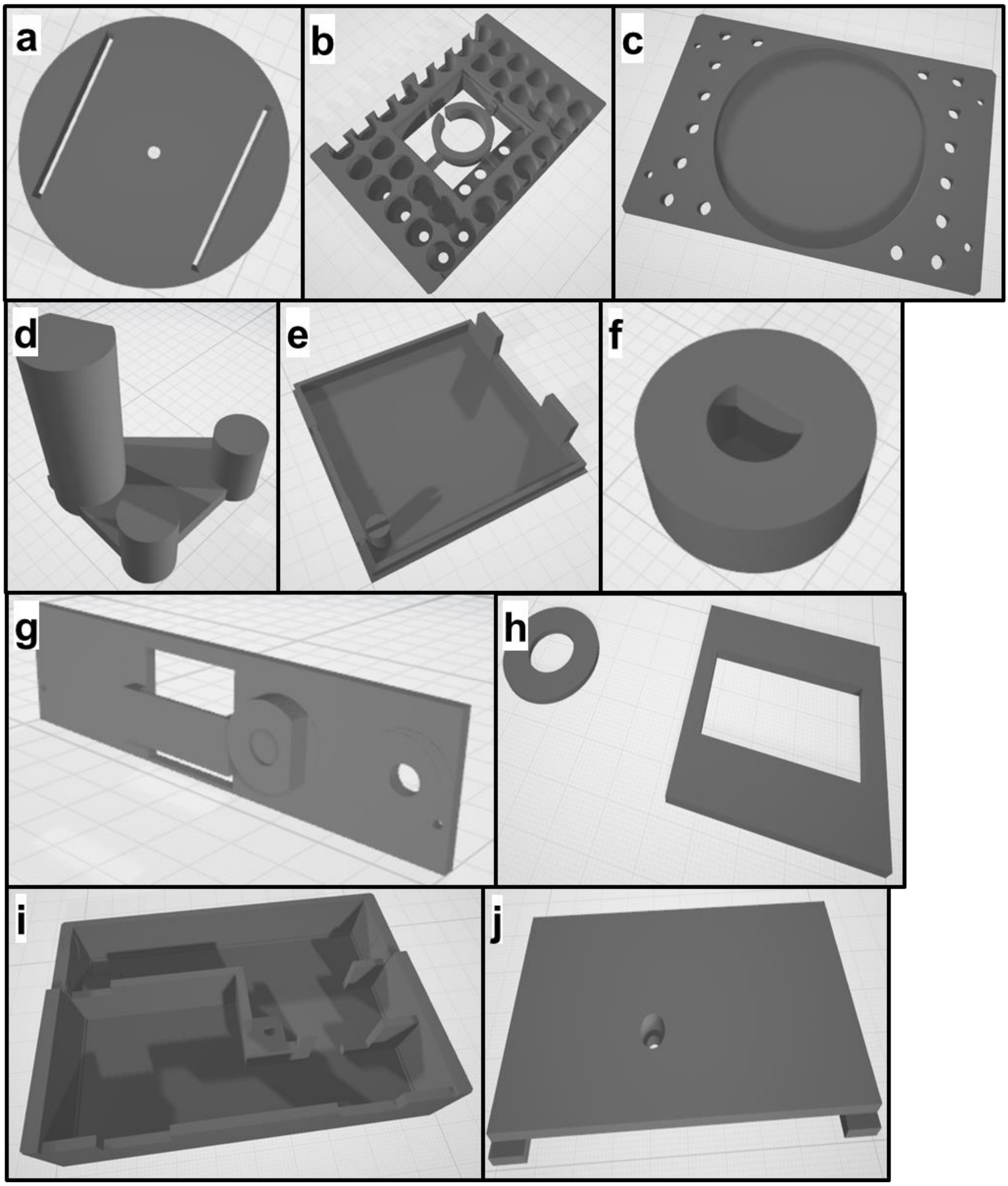

Figure S2: Snapshots of the individual 3D printed components listed in Supplementary Note 5. (a) disc.stl. (b) holder.stl. (c) coverdisc.stl. (d) sensor.stl. (e) covermotor.stl. (f) knob.stl. (g) display.stl. (h) bezel.stl. (i) control.stl. (j) covercontrol.stl.

Supplementary Note 6. Circuit board design and electronics

Circuit design is given in Figure S3 and the components list is given in Table S3.

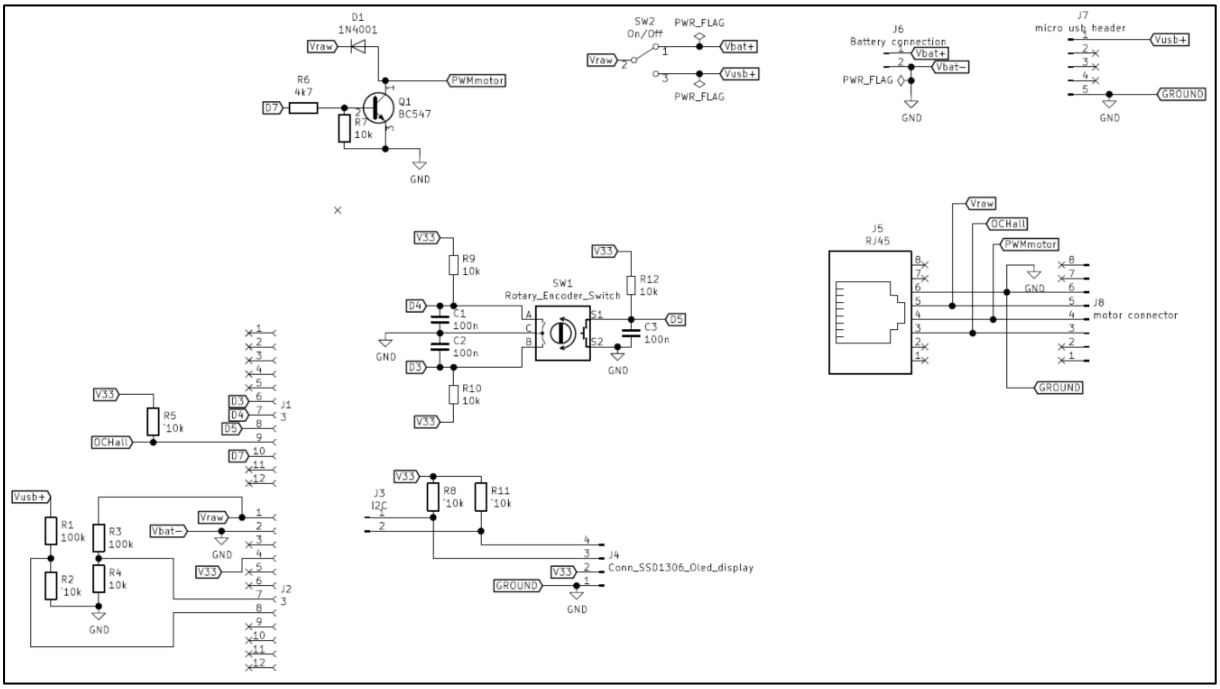

Figure S3: Printed circuit board (PCB) design.

Table S3: List of electronic and other components that are part of the PCB (Figure S3).

Reference	Value	Footprint	Part	Description
Q1	BC547	TO_SOT_Packages_THT:TO-92_Molded_Narrow_Reverse	BC547	0.1A Ic, 45V Vce, Small Signal NPN Transistor, TO-92
R6	4k7	Resistors_SMD:R_1206_HandSoldering	R	Resistor
R7	10k	Resistors_SMD:R_1206_HandSoldering	R	Resistor
SW1	Rotary_Encoder_Switch	footprintlib:Rotary_Encoder_Switch_Vertical	Rotary_Encoder_Switch-Device	
J1	3	Pin_Headers:Pin_Header_Straight_1x12_Pitch2.54mm	Conn_01x12_Female	Generic connector, single row, 01x12, script generated (kicad-library-utils/schlib/autogen/connector/)
J2	3	Pin_Headers:Pin_Header_Straight_1x12_Pitch2.54mm	Conn_01x12_Female	Generic connector, single row, 01x12, script generated (kicad-library-utils/schlib/autogen/connector/)
R3	100k	Resistors_SMD:R_1206_HandSoldering	R	Resistor
R4	10k	Resistors_SMD:R_1206_HandSoldering	R	Resistor
J5	RJ45	footprintlib:RJ45_8_REV	RJ45	RJ connector, 8P8C (8 positions 8 connected)

D1	1N4001	Diodes_THT:D_DO-41_SOD81_P10.16mm_Horizontal	1N4001	50V 1A General Purpose Rectifier Diode, DO-41
C2	100n	Capacitors_SMD:C_0805_HandSoldering	C_Small	Unpolarized capacitor, small symbol
C1	100n	Capacitors_SMD:C_0805_HandSoldering	C_Small	Unpolarized capacitor, small symbol
C3	100n	Capacitors_SMD:C_0805_HandSoldering	C_Small	Unpolarized capacitor, small symbol
R9	10k	Resistors_SMD:R_1206_HandSoldering	R_Small	Resistor, small symbol
R10	10k	Resistors_SMD:R_1206_HandSoldering	R_Small	Resistor, small symbol
R12	10k	Resistors_SMD:R_1206_HandSoldering	R_Small	Resistor, small symbol
SW2	On/Off	footprintlib:spdt switch	SW_SPDT	Switch, single pole double throw
J6	Battery connection	Pin_Headers:Pin_Header_Straight_1x02_Pitch2.54mm	Conn_01x02_Male	Generic connector, single row, 01x02, script generated (kicad-library-utils/schlib/autogen/connector/)
J8	motor connector	Pin_Headers:Pin_Header_Straight_1x08_Pitch2.54mm	Conn_01x08_Male	Generic connector, single row, 01x08, script generated (kicad-library-utils/schlib/autogen/connector/)
R1	100k	Resistors_SMD:R_1206_HandSoldering	R	Resistor
R2	10k	Resistors_SMD:R_1206_HandSoldering	R	Resistor
J7	micro usb header	Pin_Headers:Pin_Header_Straight_1x05_Pitch2.54mm	Conn_01x05_Male	Generic connector, single row, 01x05, script generated (kicad-library-utils/schlib/autogen/connector/)
R5	10k	Resistors_SMD:R_1206_HandSoldering	R	Resistor
J3	I2C	Pin_Headers:Pin_Header_Straight_1x02_Pitch2.54mm	Conn_01x02_Male	Generic connector, single row, 01x02, script generated (kicad-library-utils/schlib/autogen/connector/)
R8	10k	Resistors_SMD:R_1206_HandSoldering	R	Resistor
R11	10k	Resistors_SMD:R_1206_HandSoldering	R	Resistor
J4	Conn_SSD1306_Oled_display	Pin_Headers:Pin_Header_Straight_1x04_Pitch2.54mm	Conn_01x04_Male	Generic connector, single row, 01x04, script generated (kicad-library-utils/schlib/autogen/connector/)
