# Supplementary material for: Automated device for continuous stirring while sampling in liquid chromatography systems
Source: Commun Chem. 2020 Dec 3;3:180. doi: 10.1038/s42004-020-00427-5 (PMC9814086; doi:10.1038/s42004-020-00427-5)
Supplement: Supplementary file 2 — Description of Additional Supplementary Files [file 42004_2020_427_MOESM2_ESM.pdf]

Description of Additional Supplementary Files

File Name: Supplementary Video 1

Description: Animation of UPLC traces during a kinetics experiment.

File Name: Supplementary Video 2

Description: Video recordings of stirring in different positions in the device, at 200 and 1000 rpm.

File Name: Supplementary Dataset 1

Description: Data behind Fig. 3 and Fig. 4.

File Name: Supplementary Dataset 2

Description: STL files required for 3D printing the device.

File Name: Supplementary Dataset 3

Description: ARDUINO firmware program code.
